# Fractionation of Regenerated Silk Fibroin and Characterization of the Fractions

**DOI:** 10.3390/molecules26206317

**Published:** 2021-10-19

**Authors:** Masaaki Aoki, Yu Masuda, Kota Ishikawa, Yasushi Tamada

**Affiliations:** Faculty of Textile Science and Technology, Shinshu University, Tokida 3-15-1, Ueda 386-8567, Nagano, Japan; 19hs101f@shinshu-u.ac.jp (M.A.); nekutai0820@gmail.com (Y.M.); 21fs703h@shinshu-u.ac.jp (K.I.)

**Keywords:** ammonium sulfate, fractionation, molecular weight, nanofiber, porous structure, silk fibroin

## Abstract

The molecular weight (MW) of regenerated silk fibroin (RSF) decreases during degumming and dissolving processes. Although MW and the MW distribution generally affect polymer material processability and properties, few reports have described studies examining the influences of MW and the distribution on silk fibroin (SF) material. To prepare different MW SF fractions, the appropriate conditions for fractionation of RSF by ammonium sulfate (AS) precipitation process were investigated. The MW and the distribution of each fraction were found using gel permeation chromatography (GPC) and SDS-polyacrylamide electrophoresis (SDS-PAGE). After films of the fractionated SFs formed, the secondary structure, surface properties, and cell proliferation of films were evaluated. Nanofiber nonwoven mats and 3D porous sponges were fabricated using the fractionated SF aqueous solution. Then, their structures and mechanical properties were analyzed. The results showed AS precipitation using a dialysis membrane at low temperature to be a suitable fractionation method for RSF. Moreover, MW affects the nanofiber and sponge morphology and mechanical properties, although no influence of MW was observed on the secondary structure or crystallinity of the fabricated materials.

## 1. Introduction

Recently, silk has been examined specifically for its application as a biomaterial in medicine, especially in the tissue engineering field, due to the biosafety it has exhibited during its long and extensive use for surgical sutures [1]. Although silk fiber has been reported to be an excellent textile material for practical use as a surgical scaffold in regenerative medicine [2], many studies have examined the fabrication of silk proteins beyond silk fibers to include films [3], gels [4], sponges [5], and nanofibers [6] adapted for medical use. Silk produced by the silkworm *Bombyx mori* is comprised of proteins of two types, fibroin and sericin, which are used to construct cocoons as the main frame fiber and glue, respectively [1]. Usually the sericin protein layer, which is regarded as triggering inflammation and foreign body reactions, has been removed before any medical application by degumming through boiling in a weak alkali solution such as Na_2_CO_3_ [7]. Silk fibroin (SF) secreted into silk glands was originally a heterodimer protein of heavy-chain and light-chain molecules of about 350 kDa and 26 kDa molecular weight (MW), respectively [8,9]. However, the molecular size of regenerated silk fibroin (RSF) reportedly decreases and disperses during degumming because of heat and alkaline hydrolysis, resulting in the reduction and distribution of MW [10]. Furthermore, the degummed SF is also dissolved in a solvent such as calcium nitrate/methanol [11], CaCl_2_/H_2_O/EtOH mixed solution [12], and LiBr aqueous solution [13] for RSF solution preparation. The dissolution process reportedly induces the breakdown of SF molecules [9]. Therefore, the RSF in the solution must be the cause of the reduction and distribution of the MW.

Actually, MW and its distribution are well known to influence the mechanical, thermal, and rheological properties of polymer materials [14,15]. The mechanical properties of regenerated cellulose fibers are positively correlated with the increased MW of cellulose [16]. Reportedly, the MW distribution strongly influences the tensile properties of cellulose fiber [17]. The thermal and rheological properties of polyethylene blends with bimodal MW distributions were clarified by blending a high-MW polyethylene and low-MW polyethylene in different ratios [18]. The melting point and crystallization temperature were found to be dependent on MW and the MW distribution of blended polyethylene. The correlation between dynamic rheological parameters and the MW distribution of blended polyethylene was confirmed. Further polymer surface properties were also affected by MW and the polymer distribution. The surface molecular motion of monodispersed and polydispersed polystyrene (PS) indicated that a surface of less than 30 kDa monodispersed PS film was more active than that of higher MW PS film and indicated that the surface of polydispersed PS film was less active than monodispersed PS film at a similar MW [19]. The MW distribution influenced the phase stability of a polymer mixture. A polymer blend of mixed polymers having a narrow MW distribution was reported to show phase stability compared to polymers with a broad MW distribution [20]. A recent report described that the thermo-responsive transition of poly(N-isopropylacrylamide) was affected by MW and the distribution of MW [21]. The processability of polymer materials for thermal and rheological properties of the polymer melt or solution is also reflected by the MW and its distribution [22].

Many reports have described studies of the fabrication of SF materials with various forms using RSF, but few reports have described studies specifically examining the influence of MW and its distribution on SF material properties and processability. For the fabrication of nanofiber nonwoven mats, the influence of MW on spinnability for electrospinning and the properties of electrospun SF from the aqueous solution [23] and formic acid solution [24] as the dope was studied. The importance of MW for spinnability and the expression of properties such as mechanical strength and fiber diameter were elucidated in that study. The formation of microspheres using RSF with different MW distributions was investigated. A narrow range of MW distribution was found to promote microspheres with better shapes than a broad range of MW distribution [25]. The physical properties and structures of a SF hydrogel induced by ultrasonication treatment were studied using various MW and MW dispersion SF. One study found MW to be a crucially important condition for constructing a microscopic structure that can influence physical properties [26]. For those studies, the preparations of RSFs with different MW and MW distributions were performed by changing the degumming or dissolving conditions, such as time, temperature, and solvents, or alkaline hydrolysis of SF. However, these SFs are not strictly of the same origin, because the preparation conditions, such as degumming and dissolving, mutually differ. For that reason, the possibility of changing the structures and properties cannot be denied. To allay that concern, fractionation of MW-dispersed SF can be an appropriate method of obtaining SFs with different MW and MW distributions, despite having the same origin.

Several methods for MW fractionation of proteins are well known, including gel permeation chromatography (GPC), ultrafiltration, and ultracentrifugation. Among them, ammonium sulfate (AS) precipitation is recognized as a rapid, mild, inexpensive, and high-yield method. The first report of the precipitation of proteins using salts including AS was more than a hundred years ago [27,28]. Recently, precipitation with AS was chosen for the study of the fractionation of equine antivenom IgG [29] and gelatin from bighead carp [30]. Although AS precipitation was used to purify proteins in posterior silk glands to analyze the SF molecules [31], no report has described the MW fractionation of RSF.

The objective of this study was to elucidate the influences of MW on the processability and material properties of SF. First, the appropriate conditions for the fractionation of RSF by the AS precipitation process were investigated. Then, the fractionated SFs were analyzed using GPC, SDS-PAGE, and amino acid composition. Cast films of the fractionated SFs were formed and characterized by their secondary structure, surface properties, and cell proliferation. Furthermore, to evaluate the processability of the fractionated SFs, a nanofiber nonwoven mat was fabricated by electrospinning. A 3D porous sponge was produced using the freeze–thaw method with the fractionated SF aqueous solution. They were analyzed to elucidate their structures and mechanical properties.

## 2. Results and Discussion

### 2.1. Influences of Methods on SF Fractionation

**Figure 1:**
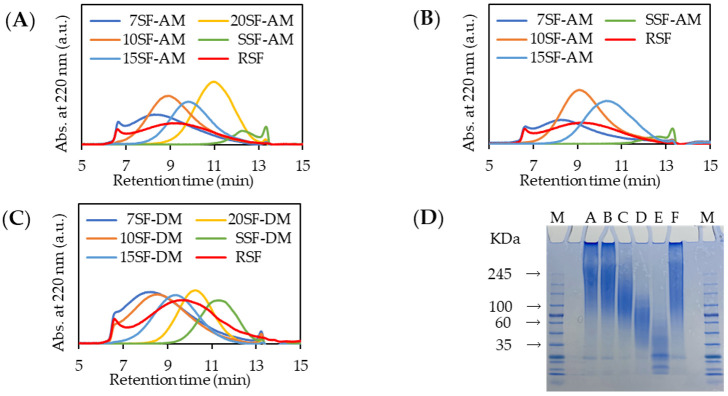
GPC elution profiles: (**A**) fractionated SFs by AM at 4 °C, (**B**) fractionated SFs by AM at 37 °C, (**C**) fractionated SFs by DM; (**D**) SDS-PAGE analysis of fractionated SFs by DM; Lane A, 7SF-DM; B, 10SF-DM; C, 15SF-DM; D, 20SF-DM; E, SSF-DM; F, RSF; and M, molecular weight marker.

Figure 1 shows the GPC elution profiles of fractionated SF by the direct addition of AS (AM, addition method) at 4 °C (A) and at 37 °C (B). The fractionated SF materials are designated as 7SF-AM, 10SF-AM, 15SF-AM, and 20SF-AM, respectively, according to the saturated concentration of AS for fractions 7%, 10%, 15%, and 20% and the SSF-AM for the fraction which was the retrieved supernatant for centrifugation of the 20% fraction.

As the profiles show, the temperature affected the SF fractionation. Actually, 20SF-AM was fractionated at 4 °C, but this result was not obtained at 37 °C. Because the solubility of protein generally depends on the temperature, we inferred that the different fractionation profiles based on temperature derived from the protein solubility [32]. 20SF-AM might be difficult to precipitate at 37 °C because of its higher solubility than at 4 °C.

During the addition of the AS powder directly into the RSF solution, avoiding a partially higher AS concentration portion in the solution against the expected AS concentration until complete dissolution was difficult. We performed a dialysis method (DM) [33] for fractionation in which the RSF aqueous solution was put into a dialysis membrane, with the tube immersed in the AS solution at each saturated concentration. Then the precipitate appearing in the dialysis membrane was collected by centrifugation, as described in the Experimental section. The GPC elution profile is presented in Figure 1C; “DM” was added to each SF designation.

Fractionation was performed more clearly than by addition method (AM). The number-averaged molecular weight (M_n_), weight-averaged molecular weight (M_w_), polymer dispersion index (PDI), and yields of the respective fractionated SFs by both methods at 4 °C, which were calculated with MW standards of pullulan, are presented in Table 1. Comparison of both methods showed the MW (M_n_ and M_w_) and PDI of every fractionated SF to be similar. However, smaller deviation at 7% and 10% fractionations on the DM than on AM was observed. A higher concentration than the expected saturation concentration occurred partially in the SF solution by adding solid AS directly. The lower MW SF fractions were precipitated at the high AS concentration. However, because the stable saturated concentration can be maintained through fractionation by the DM, the appropriate MW fractions of SF were precipitated at the saturated concentration with high reproducibility. Higher total yield was obtained using the DM than the AM, as shown in Table 1. Taken together, these findings indicate DM at 4 °C as an appropriate process for RSF fractionation. The DM was used for additional experiments in this study.

Using SDS-PAGE, we analyzed the MW and MW distributions of the fractionated SF for the respective saturated concentrations using DM. The results are shown in Figure 1D. The SDS-PAGE profile indicates successful fractionation. The average MWs, as the center of the smear band of each fractionated SF, were estimated as 245, 245, 100, 60, and 35 kDa, respectively, for 7SF-DM, 10SF-DM, 15SF-DM, 20SF-DM, and SSF-DM. The MWs estimated using SDS-PAGE were higher than those estimated using GPC because the MW standards used for estimation of MW differed for SDS-PAGE and GPC, which were, respectively, protein and polysaccharide.

The amino acids specifically examined for the determination of the amino acid composition in each fractionated SF for this study were Gly, Ala, and Ser + Tyr. These amino acids were chosen as the major and characteristic amino acids of SFs. The amounts of Gly and Ala were normalized by those of Ser + Tyr as 1; the ratio of the amino acid composition of each fractionated SF is presented in Table 2.

No significant difference of the amino acid composition among 7SF-DM, 10SF-DM, and 15SF-DM was found. The ratios were similar with RSF, but a slightly higher content of Gly was found in 20SF-DM. Actually, SF has the unique repeated sequence (Gly-Ala-Gly-Ala-Gly-Ser/Try) in the H-chain of SF [34]. The sequence is known to form a crystal structure by β-sheet conformation [35,36,37,38]. Therefore, 7SF-DM, 10SF-DM, and 15SF-DM were expected to maintain the molecular structure with RSF, except for the MW.

### 2.2. Characterization of Films from Fractionated SFs

#### 2.2.1. Surface Properties

The water contact angle of the fractionated SF films coated onto the PVC substrate was measured. The average water contact angles of 7SF-DM, 10SF-DM, 15SF-DM, 20SF-DM, and RSF were determined, respectively, as 63.7 ± 1.1°, 61.5 ± 1.1°, 62.3 ± 1.4°, 69.8 ± 3.5°, and 63.3 ± 1.2°. No significant difference was found among the fractionated SF films and RSF films, except for 20SF-DM. A slightly higher contact angle on 20SF-DM film might be explained by the amino acid composition of 20SF-DM, which has abundant hydrophobic amino acids Gly and Ala, as presented in Table 2. The dependence of MW on the water contact angle using coated films of different MW SF prepared by changing the degumming condition was reported [10]. The results presented the contact angle of a lower MW SF film as lower. The authors explained the results by the lower β-sheet contents in lower MW SF film. As described hereinafter, because no significant difference in β-sheet contents was found among the fractionated SFs, the higher contact angle of 20SF-DM film is inferred to derive from the abundant Gly and Ala in 20SF-DM.

**Figure 2:**
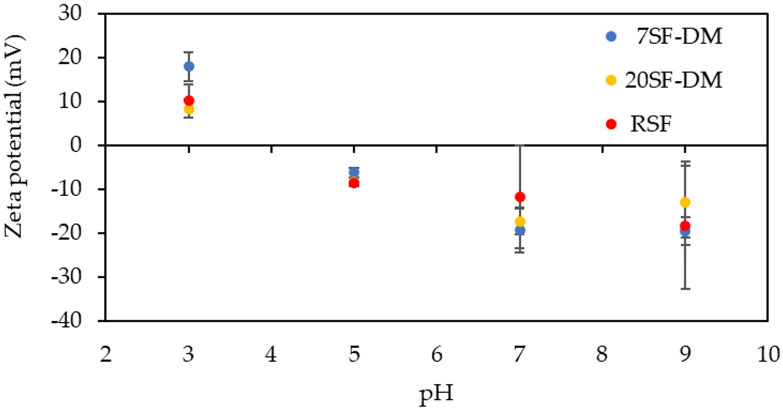
Zeta potential of fractionated SF-coated films.

The zeta potential of 20SF-DM film as another surface property was measured and compared with the 7SF-DM and RSF film. Results obtained at pH 3, 5, 7, and 9 are presented in Figure 2. No clear influence of MW on zeta potential was found. This result indicates that the charged amino acid ratio of 20SF-DM is similar to that of RSF and the other fractionated SFs.

#### 2.2.2. Secondary Structure

**Figure 3:**
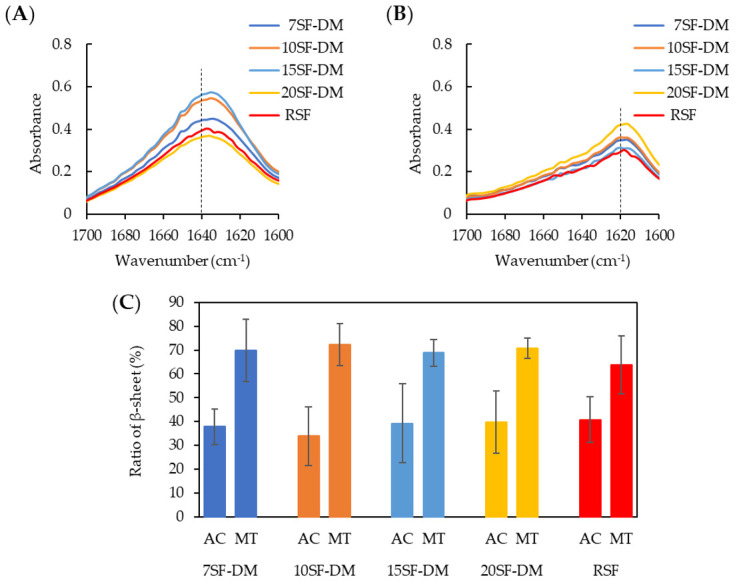
Amide I region in ATR-FTIR spectra of fractionated SF films: (**A**) as-cast films and (**B**) after methanol-treated films. Here, 1640 cm^−1^ and 1620 cm^−1^, which are assigned for random structure and β-sheets, are marked by dotted lines. (**C**): Ratio of β-sheet for as-cast (AC) and methanol treatment (MT) films.

The influence of MW on the structure of fractionated SF films was evaluated using ATR-FTIR measurements. Figure 3 depicts the spectrum of as-cast (A) and methanol-treated (B) fractionated SF films at the amide I region. As shown in Figure 3A,B, no difference in spectra was found among the fractionated SF and RSF films. The amide I peak reflects the secondary structure of the protein. The 1640 cm^−1^ and 1620 cm^−1^ peaks are attributed, respectively, to random and β-sheet structures [39]. All fractionated SF films were able to change their structure to the β-sheet structure by methanol treatment for insolubilizing, as is reported for SF films [40]. Figure 3C presents the β-sheet in the fractionated SFs films as-cast and after methanol treatment by estimation from the spectra [39]. No significant difference was found in the secondary structure of the cast films among fractionated SFs and RSFs. These results indicate that the MW of SF is unrelated to structural formation in the fractionated SF film within the range of MW examined for this study.

#### 2.2.3. Cell Proliferation Test

**Figure 4:**
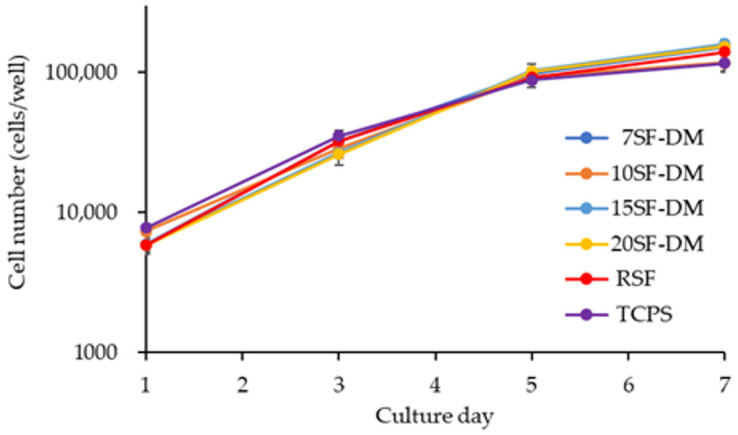
NIH3T3 cell proliferation on the coating film of the fractionated SFs and TCPS. The cell number is shown on a logarithmic scale.

To confirm the influence of MW on SF biocompatibility, a cell proliferation test was performed on the coated film of the fractionated SFs. The cell growth curve is shown in Figure 4. Cells can proliferate on all fractionated SFs films. The doubling times calculated at the logarithmic phase of cell growth were 23.6, 26.1, 23.4, 23.3, 24.2, and 27.4 h, respectively, on 7SF-DM, 10SF-DM, 15SF-DM, 20SF-DM, RSF, and TCPS. This result indicates no influence of SF MW on cell proliferation within the range of MW in this study.

### 2.3. Fabrication of Fractionated SFs

Many reports have described SF fabrication [41,42,43], but few [44] have presented consideration and discussion of the influence of MW on the fabrication processes and properties of SF materials. To evaluate the effects of MW on SF fabrication, a nanofiber nonwoven mat and 3D porous sponge were fabricated from the fractionated SF aqueous solution. Because large amounts of SFs are necessary to fabricate the SF materials, we selected two AS saturated concentrations for the fractionation of SF to obtain the SFs of different MWs: 7 and 20%, designated, respectively, as 7SF-DM2 and 20SF-DM2. From the GPC profiles of the fractionated SFs (Supplemental Figure S1), the peak MWs of 7SF-DM2 and 20SF-DM2 were estimated, respectively, as 150,000 and 85,000. They are well-separated higher and lower than RSF (peak MW; 120,000). The aqueous solution viscosity of each fraction at 8.0% (*w*/*v*) concentration was the following: 18.3 ± 0.5, 14.3 ± 1.4, and 16.9 ± 0.6 mPa·s, respectively, for 7SF-DM2, 20SF-DM2, and RSF. The viscosity of the fractionated SF solution was dependent on its MW.

#### 2.3.1. Nanofiber Nonwoven Mat

**Figure 5:**
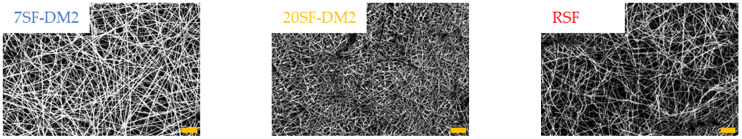
SEM images of electrospun nanofiber nonwoven mat from fractionated SF. Scale bar = 10 μm.

**Figure 6:**
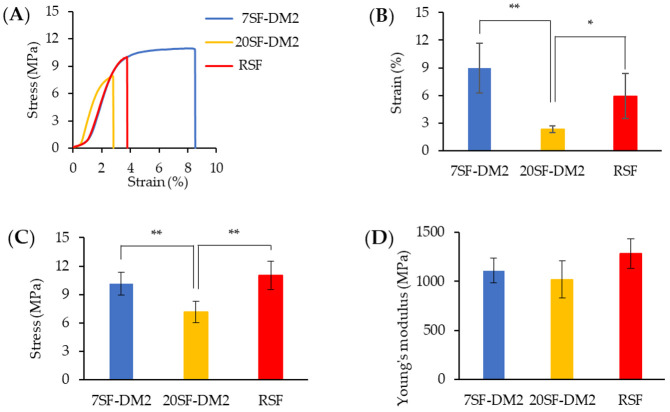
Tensile tests of SF nanofiber nonwoven mat electrospun from fractionated SF aqueous solution: (**A**) Typical stress–strain curve; (**B**) Influence of MW of SFs on strain at breaking (** *p* < 0.01, * *p* < 0.05, *n* = 5 by Tukey–Kramer test); (**C**) Influence of MW of SFs on stress at breaking (** *p* < 0.01, *n* = 5 by Tukey–Kramer test); (**D**) Influence of MW on Young’s modulus. No significant differences among fractions and RSF were found by Tukey–Kramer test (*n* = 5, *p* > 0.05).

One report has described SF nanofiber nonwoven mats from all aqueous RSF solutions as the spinning solution by the electrospinning process [45]. Both fractionated SF aqueous solution, 7SF-DM2 and 20SF-DM2 were available for a nanofiber mat by electrospinning, similarly to RSF. The fiber morphologies observed by SEM are shown in Figure 5. No significant difference of fiber diameters was found among SFs and RSF, and the diameter was estimated at around 400 nm. Although the fiber morphology of the 7SF-DM2 nonwoven mat was the same as that of RSF, several beads appeared on the fibers of the 20SF-DM2 nonwoven mat. Kishimoto et al. reported that beads were induced in the electrospun SF nonwoven mat by lower MW SF [23]. The β-sheet contents and the crystallinity index among the fractionated SFs and RSF nanofibers estimated by ATR-FTIR spectrum (Supplemental Figure S2) were observed. No significant difference was found. These results show good agreement with the fractionated SF film results, as described Section 2.2.2. The mechanical properties of the fractionated SF nanofiber nonwoven mat as measured by the tensile test and, according to the strain–stress curve (A), and the breaking stress (B), breaking strain (C), and Young’s modulus (D) are shown in Figure 6. Although the Young’s modulus of the 20SF-DM2 nanofiber was the same as that of the 7SF-DM2 and RSF nanofiber, the breaking stress and strain of the 20SF-DM2 nanofiber were significantly lower. This finding shows good agreement with results reported [22] for the dependence of MW on the mechanical properties of SF nanofibers. The breaking strain of the 7SF-DM2 nanofiber was much higher than that of 20SF-DM2 and even of the RSF nanofiber. These results show that the 7SF-DM2 nanofiber toughness is superior to that of RSF nanofibers. These results indicate that the MW of SF is an important factor for fabrication by electrospinning and an important factor affecting the mechanical properties of the resulting nanofiber nonwoven mat.

#### 2.3.2. Porous 3D Structure (Sponge)

**Figure 7:**
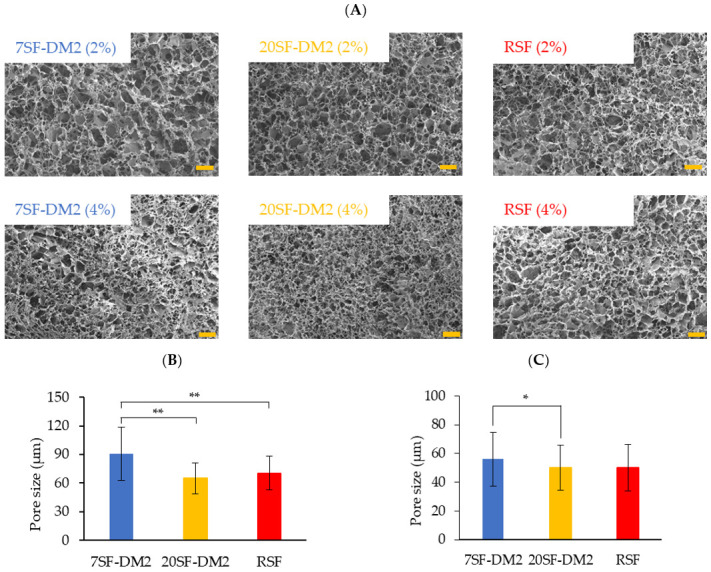
(**A**) SEM images of sponges fabricated from the fractionated SFs. Scale bar = 100 μm. Significance was inferred from *t*-test results, shown as ** *p* < 0.01 and * *p* < 0.05 (*n* = 50). Pore sizes of sponges fabricated at (**B**) 2% (*w*/*v*) and (**C**) 4% (*w*/*v*) of SF aqueous solution. Pore size was measured using Image J from SEM images.

The porous 3D structure (sponge) of SF can be fabricated by freeze–thaw processing using RSF aqueous solution mixed with a small amount of water-miscible organic solvent such as DMSO [46]. Both fractionated SF aqueous solutions were available to fabricate SF sponges by freeze–thaw processing. The pore structure was observed by SEM as presented in Figure 7A. No apparent difference of the pore shape was found between the fractionated SFs and RSF. Figure 7B,C present the average pore size as measured using SEM images. The 7SF-DM2 sponge pores were found to be markedly larger than those of the 20SF-DM2 sponge. We inferred that the pores in the 20SF-DM2 sponge became smaller than those of 7SF-DM2, as follows. The pore size of the SF sponge fabricated using the freeze–thaw process is determined by the size of the ice crystals grown during the freezing time. The ice crystals can grow to larger sizes when the ice crystallization heat is removed more slowly. The lower MW fraction SF molecules can dissolve at a higher concentration in the aqueous solution than the higher MW fraction SF molecules. Because the specific heat capacity of the aqueous solution is lower at a higher solute concentration, the heat of ice crystallization in the lower MW fractionated SF solution can escape faster than in the higher MW fractionated SF solution. Pore sizes of the RSF sponge were observed between 7SF-DM2 and 20SF-DM2. These results indicate that the MW of the fractionated SF affects the pore size of SF sponge, although the influence was a little.

The ATR-FTIR spectra of the fractionated SF sponges were measured. Similar spectra with a peak at 1625 cm^−1^ were obtained (Supplemental Figure S3). The β-sheet structure contents were estimated at around 61–65% of 2% (*w*/*v*) and as around 65–66% of 4% (*w*/*v*) fractionated SF sponges. The MW did not influence the secondary structure of the fractionated SF sponge as the film and nanofiber did.

The compressive modulus of the fractionated SFs sponges is presented in Table 3. No significant difference was found among the SFs and RSF sponges at 2% concentration, but in the case of the 4% sponge, the 20SF-DM2 sponge showed a markedly higher compressive modulus than the others. We inferred that the higher compressive modulus of the 4% 20SF-DM2 sponge might derive from the smaller pore size, as shown in Figure 7. For the 2% sponge, because the SF content in the sponge wall is too small to detect the mechanical difference, the apparent compressive modulus of 20SF-DM2 sponge might be measured similarly to that of the 7SF-DM2 sponge.

## 3. Materials and Methods

### 3.1. Preparation of RSF Aqueous Solution

Degummed silk thread (*Bombyx mori*) was donated by Dr. Takabayashi (National Institute of Agrobiological Science, Okaya, Japan). *Bombyx mori* cocoons were obtained from Art Co. Ltd., Gunma, Japan and were degummed as described in an earlier report. The degummed silk was dissolved in 9 M LiBr (Fujifilm Wako Pure Chemical Corp., Tokyo, Japan) and was dialyzed for 3 days with reverse osmosis (RO) water to prepare an RSF aqueous solution. Then, the RSF aqueous solution was concentrated by air-drying at room temperature (r.t., 25 °C). The insoluble aggregations in the concentrated solution were removed by centrifugation. The RSF aqueous solution concentration was found by weight measurement after drying.

### 3.2. Fractionation with Ammonium Sulfate (AS)

Fabrication of SF by AS precipitation was performed by (1) AS powder addition (addition method (AM)) and (2) dialysis in AS solution (dialysis method (DM)) [33].

For the addition method (AM), RSF aqueous solution was diluted with RO water at 1.5% (*w*/*v*) concentration. The volume was adjusted to 100 mL with AS powder to become 7, 10, 15, and 20% of the saturated concentration. It was added gradually to the RSF aqueous solution under stirring. After stirring was continued for 1 h at r.t., the solution was left to stand overnight at 4 °C or 37 °C. The precipitations at each AS saturated concentration were collected by centrifugation (10,000 rpm × 30 min). Then, the supernatant was used for fractionation continuously at a higher AS saturated concentration.

For the dialysis method (DM), RSF aqueous solution was diluted with RO water at 1.5% (*w*/*v*) concentration. After the volume was adjusted to 100 mL, it was placed in a dialysis membrane (MWCO: 12,000–14,000 Da; AS One Corp., Osaka, Japan). The dialysis membrane was immersed into 500 mL of AS 7% saturation concentration solution at first. The dialysis solution was incubated for more than 12 h at 4 °C or 37 °C. The precipitate on the dialysis membrane was corrected by centrifugation. Then the supernatant was placed in a new dialysis membrane. Furthermore, the dialysis membrane was immersed into 10% AS saturated concentration solution. The precipitate was collected. This fractionation process was repeated at 15 and 20% AS saturation concentration. The obtained precipitations were washed using RO water and were freeze-dried for additional experiments.

### 3.3. Fabrication of SF

A film was formed by casting the 0.5% (*w*/*v*) fractionated SF aqueous solution onto a polystyrene dish (*Φ* 55 × 17; AS One Corp., Osaka, Japan), followed by incubation at 50 °C. The films were soaked into 80% (*v*/*v*) methanol for insolubilization and were dried at 50 °C.

The coated film was prepared by incubation of 0.5% (*w*/*v*) SFs aqueous solution on a polyvinyl chloride plate at r.t. for 30 min. Then the solution was removed. The coated film was soaked into 80% (*v*/*v*) methanol for insolubilization and dried at 50 °C.

The nanofiber nonwoven mat was fabricated by electrospinning. The electrospinning was performed using a solution type electrospinning system (Nanon-3; MECC Co., Ltd., Fukuoka, Japan) according to conditions reported earlier. In brief, the fractionated SF aqueous solution was diluted to 8% (*w*/*v*) concentration with RO water and adjusted pH to 10.5 with 5 M NaOH (Fujifilm Wako Pure Chemical Corp., Tokyo, Japan) with ethanol added (99.5% (*v*/*v*); Fujifilm Wako Pure Chemical Corp., Tokyo, Japan) to 3% (*v*/*v*) concentration, then stirred at r.t. Electrospinning was performed at 18 kV on 20 cm distance between the spinneret and collector. The electrospun nonwoven mat was incubated for 30 min in water vapor under 37 °C for insolubilization.

According to processes described for an earlier report, 3D porous structures (sponges) were fabricated by freeze–thaw processing. In brief, the SF aqueous solution concentration was adjusted to 2% (*w*/*v*) and 4% (*w*/*v*); DMSO (Fujifilm Wako Pure Chemical Corp., Tokyo, Japan) was mixed at 1% (*v*/*v*) concentration. The solution was placed in an aluminum mold and was frozen to −20 °C under programmed control. Then it was thawed at r.t.

### 3.4. Determination of Molecular Weight (MW)

The fractionated SFs were dissolved in 9 M LiBr solution and were then dialyzed against RO water. The fractionated SF aqueous solutions were diluted to 0.1% (*w*/*v*) with an elution buffer (1/15 M pH 7.0 phosphate buffer containing 2 M urea and 0.1 M Na_2_SO_4_) for gel permeation chromatography (GPC) analysis. The sample solutions were filtered through a 0.45 μm hydrophilic PTFE membrane (Merck KGaA, Darmstadt, Germany). A GPC column (KW-804; Showa Denko K.K., Kanagawa, Japan) was used. GPC was performed using a high-performance liquid chromatograph (HPLC) system (Shimadzu Corp., Kyoto, Japan). The HPLC was operated at a flow rate of 1.0 mL/min at 30 °C. A MW standard was used (Pullulan; Showa Denko K.K., Kanagawa, Japan). Then the MW was estimated by calibration. M_n_, M_w_ and PDI were calculated, respectively, using the following equations [47,48,49].

M_n_ = Σ H_i_/Σ (H_i_/M_i_) (1)

M_w_ = Σ (H_i_ × M_i_)/Σ H_i_ (2)

PDI = M_w_/M_n_ (3)

Therein, M_i_ stands for the MW of a molecule chain calculated using Pullulan, H_i_ denotes the chromatogram heights, and i expresses a dividing point of retention.

Subsequently, SDS-PAGE was performed as follows. The fractionated SF solution in running buffer (Tris-HCl, SDS, sucrose, dithiothreitol (DTT) and bromophenol blue (BPB), E-T520L; ATTO Corp., Tokyo, Japan) were heated at 98 °C for 5 min and were then run on a 5–20 wt% polyacrylamide gradient gel (E-T5520L; ATTO Corp., Tokyo, Japan). A molecular marker of 10–245 kDa (WSE-7020; ATTO Corp., Tokyo, Japan) was used for estimation of the MW and the distribution. Electrophoresis was performed for 75 min with PageRun-R (ATTO Corp., Tokyo, Japan) using a current of 10.5 mA. After electrophoresis, the gel was immersed in a stain solution (EzStain Aqua; ATTO Corp., Tokyo, Japan) and was then washed with RO water overnight.

### 3.5. Amino Acid Compositions Analysis

After 0.01 g of dried fractionated SFs in 6 M HCl_aq_ were treated for 18 h at 105 °C, the hydrolyzed solution was neutralized by 0.2 M sodium citric acid and filtered through a 0.45 µm filter (Hawach Scientific Co. Ltd., Shaanxi, China). The amino acid compositions were ascertained using a prominence amino acid analysis system (RF20AXS; Shimadzu Corp., Kyoto, Japan) and a Na-type amino acids mobile-phase kit (Shimadzu Corp., Kyoto, Japan). The amino acid compositions were glycine, alanine, serine, and tyrosine, which are the major amino acids in the SF molecule. They were calculated with normalization against the total concentrations of serine and tyrosine.

### 3.6. Characterizations

#### 3.6.1. FTIR

The FTIR spectra were measured using an infrared spectrometer (Prestage-21; Shimadzu Corp., Kyoto, Japan) with ATR equipment (DuraSamplIR; Smiths Detection, London, UK) in the region of 600–4000 cm^−1^ at r.t. Spectra were recorded with an accumulation of 30 scans and resolution of 4 cm^−1^. The amide I (1600–1700 cm^−1^) peaks of the FTIR spectra were decomposed and curve-fitted using software (OriginPro 8.1; OriginLab Corp., Northampton, MA, USA) for analysis of the β-sheet content.

The crystallinity index of the fractionated SF nanofiber nonwoven mat was calculated from the amide III band in the FTIR spectrum [44]. The crystallinity index was calculated using Equation (4):

Crystallinity index (%) = Absorbance at 1260 cm^−1^/Absorbance at 1235 cm^−1^ × 100 (4)

#### 3.6.2. Mechanical Tests

Tensile tests for the fractionated SF nanofiber nonwoven mats were performed using a test machine (EZ-SX; Shimadzu Corp., Kyoto, Japan) with a 5 N load cell. The sample length was set as 30 mm. The crosshead speed was 10 mm/min. The sample thickness was measured using a micrometer (Digimatic micrometer MDQ-30MX; Mitsutoyo Corp., Kanagawa, Japan) at several points. The averaged and cross-sectional areas were calculated.

The compression modulus of fractionated SF sponges were measured using a test apparatus (EZ Test EZ-S; Shimadzu Corp., Kyoto, Japan) with a 50 N load cell at 5 mm/min of compression speed. The compression modulus determined the initial slope in the stress–strain curve.

#### 3.6.3. Viscosity

Viscosity of the fractionated SF aqueous solution was measured at 20 °C using an oscillation type viscometer (VM-10A series, Viscomate; Sekonic Corp., Tokyo, Japan). The solution concentration was 0.5% (*w*/*v*). After each sample was measured three times, the results were averaged.

#### 3.6.4. Water Contact Angle

Contact angles of fractionated SF coated materials against RO water were measured using the sessile drop method with a contact angle meter (DMs-400; Kyowa Interface Science Co., Ltd., Saitama, Japan). After 2 µL of RO water was dropped onto the coated films, measurements were taken 60 times at intervals of 500 ms. The contact angle data against time were extrapolated to 0 s; the angle at 0 s was defined as the water contact angle.

#### 3.6.5. Zeta Potential

Measurements of the zeta potential for the films coated onto the glass were conducted by a zeta potential and particle size analyzer (ELSZ-2000Z; Otsuka Electronics Co., Ltd., Osaka, Japan). The buffer for the measurement was prepared as follows: NaCl_aq_ of 5 mM was added to adjust the concentration of phosphate buffer to 5 mM. Buffers of 3, 5, 7, and 9 pH were prepared with HCl_aq_ and NaOH_aq_. The particle for monitoring was diluted using these buffer solutions. The monitor dispersion for measurement was prepared.

#### 3.6.6. Scanning Electron Microscopy (SEM)

Scanning electron microscope images were taken at 10 kV (SEM: JSM-6010LA; JEOL Ltd., Tokyo, Japan) after coating with platinum. The fiber diameter of nanofiber and the diameter of the pore size of sponges were ascertained using software (ImageJ NIH, 1.53e) from SEM images.

#### 3.6.7. Cell Culture

To evaluate the cell proliferation behavior on the coated films fabricated from fractionated SF, NIH3T3 cells were used for the test. First, 5000 cells/mL/well were seeded on each sample and were incubated at 37 °C and 5% CO_2_. After 1, 3, 5, and 7 days of incubation, PBS rinsing, and addition of Triton X-100/PBS were performed similarly to the cell adhesion test described above for cell number counting on each culture day.

The number of cells was determined by the LDH activity measurement method [50]. Briefly, the LDH activity from cell lysate in Triton-×100/PBS solution was measured by NADH consumption using the change of the optical density at 340 nm. The cell number was calculated using calibration data using LDH activity against the known cell number.

## 4. Conclusions

Fractionation of SF from RSF aqueous solution was performed by precipitation of the AS solution. Fractionation with AS using a dialysis membrane at low temperature was found to be the appropriate fractionation process for SF. The fractionated SFs were characterized using GPC and SDS-PAGE. Each fractionated SF showed different MW. Amino acid analysis revealed a different composition in the lowest MW fractionated SF. The coated films formed from the fractionated SFs presented the same secondary structure, zeta potential, and cell proliferation, but the lowest MW fractionated SF coated film showed slightly greater hydrophobicity than the others. The fractionated SFs were fabricated to nanofiber nonwoven mats by electrospinning and to porous sponge structures by freeze–thaw processing, similar to non-fractionated SFs. No influence of MW on the secondary structure and crystallinity of nanofibers and sponges was observed, but MW of SF affected the morphology and mechanical properties of nanofibers and sponges. We concluded that the MW difference of SF within the range of this study is not a crucially important condition for SF fabrication.

## Figures and Tables

**Table 1:** Number-averaged molecular weight (M_n_), weight-averaged molecular weight (M_w_), polymer dispersion index (PDI) and yield ratios of precipitation at fractionation. Fractionation was repeated three times: (A) AM and (B) DM.

(**A**)								
**Sample**	**M_n_**		**M_w_**		**PDI**		**Yield (%)**	
	**Avg.**	**Std.**	**Avg.**	**Std.**	**Avg.**	**Std.**	**Avg.**	**Std.**
7SF-AM	75,000	9600	190,000	20,000	2.5	0.44	17.0	8.1
10SF-AM	70,000	8900	140,000	24,000	2.0	0.14	13.5	8.9
15SF-AM	46,000	4400	76,000	8100	1.7	0.10	8.16	8.6
20SF-AM	32,000	5600	44,000	8300	1.4	0.09	3.76	1.3
SSF-AM	15,000	6000	19,000	8700	1.2	0.08	0.98	0.56
RSF	52,000	1500	140,000	12,000	2.6	0.29		
Total							43.4	13.9
(**B**)								
**Sample**	**M_n_**		**M_w_**		**PDI**		**Yield (%)**	
	**Avg.**	**Std.**	**Avg.**	**Std.**	**Avg.**	**Std.**	**Avg.**	**Std.**
7SF-DM	66,000	2900	160,000	11,000	2.3	0.13	15.8	2.0
10SF-DM	70,000	9000	140,000	11,000	2.1	0.13	25.5	15.1
15SF-DM	51,000	11,000	82,000	15,000	1.6	0.14	11.1	3.7
20SF-DM	35,000	4300	48,000	3700	1.4	0.12	9.11	2.83
SSF-DM	17,000	4500	21,000	6400	1.2	0.06	1.66	0.63
RSF	54,000	9100	130,000	15,000	2.4	0.14		
Total							67.7	16.5

**Table 2:** Amino acid composition of fractionated SF. The values of Gly and Ala were normalized by those of Ser + Tyr as 1.

	7SF-DM	10SF-DM	15SF-DM	20SF-DM	RSF
Gly	3.48	3.33	3.80	4.72	3.52
Ala	1.64	1.57	1.72	1.96	1.68
Ser + Tyr	1.00	1.00	1.00	1.00	1.00

**Table 3:** Compressive moduli of sponges fabricated from (A) 2% (*w*/*v*) and (B) 4% (*w*/*v*) of 7SF-DM2, 20SF-DM2, and RSF solutions.

Sample	7SF-DM2, MPa	20SF-DM2, MPa	RSF, MPa
SF 2%	1.2 ± 0.09	1.1 ± 0.06	1.1 ± 0.1
SF 4%	1.3 ± 0.1	1.5 ± 0.05	1.2 ± 0.1

## Data Availability

The data presented in this study are available in the article.

## Supplementary Materials

The following are available online, Figure S1, GPC elution profiles of SFs (7SF-DM2, 20SF-DM2) fractionated by DM and RSF; Figure S2, Amide I region in ATR-FTIR spectra of electrospun nanofiber nonwoven mat from fractionated SF; Figure S3, Amide I region in ATR-FTIR spectra of sponges fabricated from (A) 2% (*w*/*v*) and (B) 4% (*w*/*v*) of 7SF-DM2, 20SF-DM2, and RSF solutions.

## Sample Availability

Sample of the regenerated silk fibroin is available from the authors.
