# Supplementary material for: Fractionation of Regenerated Silk Fibroin and Characterization of the Fractions
Source: Molecules. 2021 Oct 19;26(20):6317. doi: 10.3390/molecules26206317 (PMC8540890; doi:10.3390/molecules26206317)
Supplement: Supplementary file 1 [file molecules-26-06317-s001.zip › molecules-1421118-supplementary.pdf]

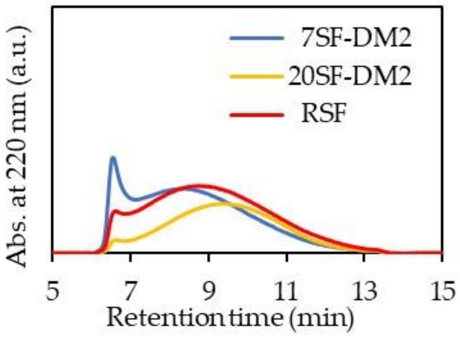

Figure S1. GPC elution profiles of SFs (7SF-DM2, 20SF-DM2) fractionated by DM with 7% and 20% of saturated concentration AS and RSF for use in fabrication experiments.

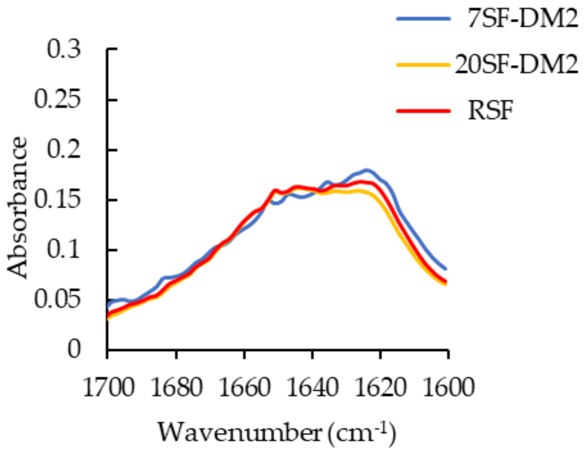

Figure S2. Amide I region in ATR-FTIR spectra of electrospun nanofiber nonwoven mat from fractionated SF.

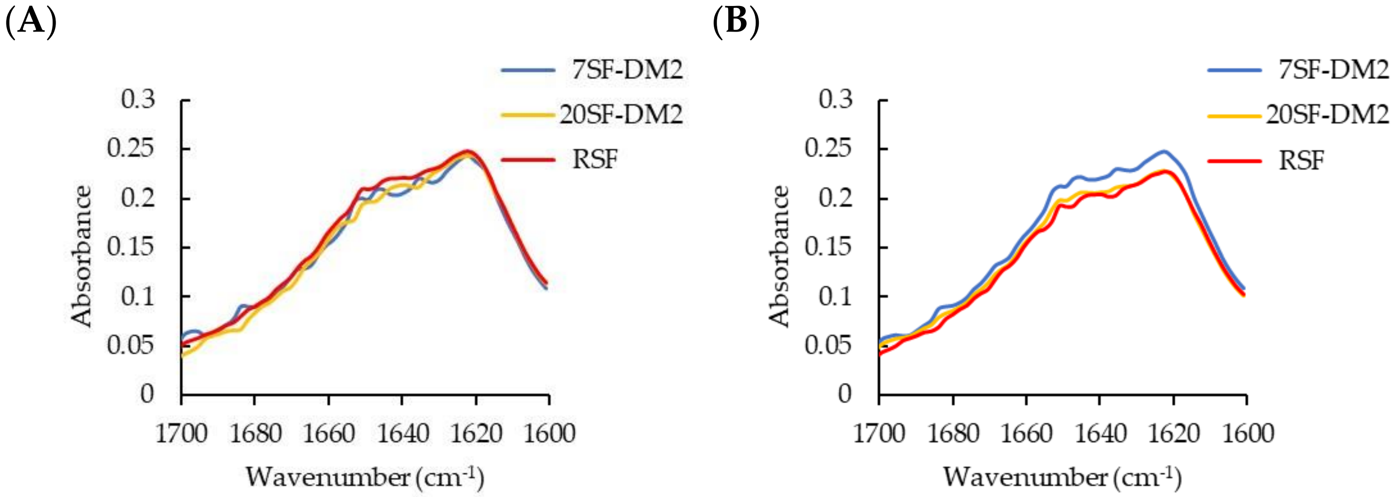

Figure S3. Amide I region in ATR-FTIR spectra of sponges fabricated from (**A**) 2% (w/v) and (**B**) 4% (w/v) of 7SF-DM2, 20SF-DM2, and RSF solutions.
